# Phosphorus fertilization enhanced overwintering, root system and forage yield of late-seeded alfalfa in sodic soils

**DOI:** 10.1038/s41598-024-67087-6

**Published:** 2024-08-05

**Authors:** Yuntao Wang, Jihong Xie, Fan Fan, Zhen Sun, Feng Yuan, Qiqi Wang, Linqing Yu, Yaling Liu, Jie Li, Lele Cui

**Affiliations:** 1 School of Grassland, Resources and Environment, Inner Mongolia Agricultural University, Hohhot, 010011 China; 2 Institute of Grassland Research, Chinese Academy of Agricultural Sciences, Hohhot, 010010 China; 3 Institute of Crop Sciences, Chinese Academy of Agricultural Sciences, Beijing, 100081 China; 4 School of Medicine, Qingdao Qiushi College, Qingdao, 266108 China; 5 National Center of Pratacultural Technology Innovation (Under Preparation), Hohhot, 010030 China; 6 School of Life Sciences, Inner Mongolia University, Hohhot, 010021 China

**Keywords:** *Medicago sativa* L., Sowing date, Phosphorus fertilization, Overwintering rate, Root traits, Forage production, Plant development, Plant morphogenesis, Plant stress responses, Salt

## Abstract

Sowing date and soil fertility are very important factors in the overwintering and production performance of alfalfa (*Medicago sativa* L.), yet there’s a knowledge gap in knowledge on how late-seeded alfalfa responds to phosphorus (P) fertilization. A field study was conducted in Inner Mongolia from 2020 to 2022 using a split-plot design. The main plots consisted of five sowing dates (31 July, 8, 16, and 24 August, and 1 September), while the subplots involved five P application rates (0, 40, 70, 100, and 130 kg P_2_O_5_ ha^−1^). Throughout the growing seasons, the overwintering rate, root traits, forage yield, and yield components were measured. The results revealed a consistent decrease in overwintering ability and productivity with the delayed sowing. This reduction in overwintering rate was mainly due to diminished root traits, while the decrease in forage yield was largely associated with a reduction in plants per square meter. However, P fertilizer application to late-seeded alfalfa demonstrated potential in enhancing the diameter of both the crown and taproot, thus strengthening the root system and improving the overwintering rate, the rate of increase ranges from 11.6 to 49%. This adjustment could also improve the shoots per square meter and mass per shoot, increasing by 9.4–31.3% and 15.0–27.1% respectively in 2 years, which can offset the decline in forage yield caused by late sowing and might even increase the forage yield. Regression and path analysis indicated that alfalfa forage yield is primarily affected by mass per shoot rather than shoots per square meter. This study recommended that the sowing of alfalfa in similar regions of Inner Mongolia should not be later than mid-August. Moreover, applying P fertilizer (P_2_O_5_) at 70.6–85.9 kg ha^−1^ can enhance the forage yield and persistence of late-seeded alfalfa. Therefore, appropriate late sowing combined with the application of P fertilizer can be used as an efficient cultivation strategy for alfalfa cultivation after a short-season crop harvest in arid and cold regions.

## Introduction

Alfalfa (*Medicago sativa* L.) known as the “Queen of the Forages”, is a perennial leguminous forage crop. It is the primary forage crop cultivated in temperate regions worldwide because of its robust vitality, high nutritional content, remarkable yields, adaptability, and wide-ranging uses^1^. Alfalfa is cultivated over 40 million hectares worldwide^2^. Meanwhile, by 2021, the cultivation area of alfalfa in China had reached 4.23 million hectares, accounting for over 40% of the total area of high-quality forage in China^3^. Alfalfa is mainly distributed in the northern, northwestern, and northeastern regions^4^. In recent years, driven by the continuous development of animal husbandry and ecological construction, the alfalfa planting area rapidly increased in China. It is imperative to highlight the alfalfa production and cultivation techniques research tailored to regional needs.

Selecting the optimal sowing date is pivotal for maximizing the utilization of climatic resources such as light, water, and heat which are vital for shoots and root growth^5^. Alfalfa can be planted in most temperate regions of China from spring to autumn. Spring sowing requires extra irrigation to mitigate the effects of spring drought. High temperatures during summer planting can impede alfalfa growth, with weed presence becoming more noticeable. As a result, alfalfa cultivation during the spring and summer demands substantial labor and resource investments for successful establishment. Autumn sowing benefits from lower weed incidence, favorable temperatures, sufficient rainfall, and high seedling emergence rates^6^. This sowing period also aligns well with the post-harvest of summer crops, integrating smoothly into the existing cropping system. Nonetheless, sowing delays can curtail the amount of radiation the crop intercepts during its establishment phase, adversely affecting root growth and shoot yields^7^. Yields from later sowing dates remain lower in the subsequent year due to increased partitioning to roots^5,8,9^. The crop's yield is approximately 20% less in their first season after establishment compared to their average yield in the second to fifth years^8^. Consequently, researching and improving late-sowing techniques for cultivating alfalfa, while ensuring the safe overwintering and subsequent year’s production performance, holds significant practical importance and applicational value.

Phosphorus (P) is considered a vital nutrient essential for plant growth and development^10^. Although alfalfa does not exhibit a high demand for P, it plays a crucial role in its growth and development processes^11^. Phosphorus plays a crucial role not only in the formation of proteins and enzymes, but also as a constituent of nucleic acid, nucleoprotein, phospholipids, ATP, and ADP^12^. Phosphorus can also enhance the activity of nitrate reductase in plant roots, which in turn facilitates nitrogen uptake^13^. Furthermore, phosphorus is instrumental in regulating and maintaining metabolic processes within plants, enabling them to adapt to stress conditions. For instance, adequate P nutrition is advantageous in enhancing the resilience of crops against cold, drought, diseases, and lodging^14^. Recent studies have shown that P application can improve root structure and increase the content of soluble sugars and proteins in alfalfa, which are crucial for its cold resistance^15^. Applying P at a depth of 15 cm from the soil surface can maximize its benefits, as it increases the content of nitrogen-containing protective substances and reduces relative conductivity under cold stress, thereby improving cold resistance^16^. Moreover, the proper application of P fertilizer has been found to increase alfalfa's dry matter yield and quality, with a significant effect on crude protein content and relative feeding value^17^.

The precipitation and temperature conditions in late summer and early autumn of Inner Mongolia present an ideal environment for alfalfa seeding. However, the constrained growth period before frost and P deficiency could hinder alfalfa's adequate development, negatively affecting its overwintering capability and forage yields in subsequent years. Previous studies about sowing time and fertilizer on alfalfa were often separate^8,18–20^, but this study combines the two factors and specifically investigates the response of late-seeded alfalfa to P fertilization. It is hypothesized that applying P fertilizer to the late-seeded alfalfa would improve its growth and development before winter, particularly by enhancing the root system, which in turn, would bolster overwintering success and yields in the following years. The objectives of this study were: (i) to determine the effects of sowing dates and P fertilizer application on alfalfa overwintering rates and root traits; (ii) to assess the influence of sowing date and P fertilizer application affect the yield and persistence of alfalfa; (iii) to identify the optimal sowing date and the corresponding P application rate for maximizing alfalfa cultivation outcomes in central Inner Mongolia regions.

## Materials and methods

### Experimental site

The experiment lasted for 3 years, starting in year 2020. It was conducted at an agro-pastoral experiment station located 35 km south of Hohhot, Inner Mongolia (40° 34′ N, 111° 45′ E, and 1050 m ASL). The study area has a semi-arid continental climate, categorized by drought, low temperatures, and windblown sand. The average annual air temperature is 5.6 °C, with an annual precipitation of approximately 400 mm, mainly concentrated in summer (June, July, and August). Before planting, soil samples were collected from a depth of 30 cm and later analyzed using a soil nutrient analyzer (Shandong Yuntang Intelligent Technology Co., Ltd., Weifang, China)^13^. The soil exhibits the following characteristics: 6.0% organic matter, 1.1 g kg^−1^ total nitrogen, 69.45 mg kg^−1^ available nitrogen, 20.5 mg kg^−1^ available phosphorus, 93.0 mg kg^−1^ available potassium, and a pH of 8.5^13^. The air temperatures and precipitation for the years 2020, 2021, and 2022 are shown in Table 1, respectively.

**Table 1:** The air temperatures (°C) and precipitation (mm) by month for each year.

Month	Temperature (°C)			Precipitation (mm)		
	2020	2021	2022	2020	2021	2022
Jan	− 8.5	− 9.5	− 6.1	3.3	5.3	2.4
Feb	− 4.0	0	− 7.2	14.5	0.1	4.2
Mar	3.2	4.5	5.5	25.1	6.7	15.2
Apr	8.5	8.5	11.5	3.7	0.1	2.7
May	16.2	15.1	16.5	29.2	23.5	26.6
Jun	20.1	20.5	23.1	73.2	19.4	67.3
Jul	21.3	23.2	23.5	89.1	104.7	80.2
Aug	20.2	20.4	21.5	68.9	171.6	47.4
Sept	14.5	17.5	16.2	25.3	54.2	30.1
Oct	5.5	7.5	7.3	23.2	10.2	23.3
Nov	− 0.5	− 1.3	0.5	0.1	5.7	0.1
Dec	− 11.2	− 6.5	− 10.2	0.1	0.3	0.3

The table temperatures were the mean of high and low temperatures.

### Treatments, experimental design and crop husbandry

The alfalfa variety used in the study was Zhongcao No. 3, known for its resistance against drought and cold stresses, as well as its moderately salt tolerant, making it suitable for dry cultivation. It has been widely adopted and cultivated in Hohhot, Baotou, Ulanqab, Ordos, Chifeng, Bayannur, and Xing’an city of Inner Mongolia. The treatment design was a factorial combination of sowing dates as the main plots and P fertilization as the subplots in a split-plot design with four replications^21^. The sowing dates were 31 July, 8, 16, and 24 August, and 1 September, denoted as T1, T2, T3, T4, and T5, respectively. The plot area was 3 × 5 m, and the sowing rate was 22.5 kg ha^−1^ with a 30 cm row spacing and 1–2 cm depth. A 1-m-wide border surrounds the experimental site was made. Phosphorus fertilizer (produced by Henan Jixin Chemical Products Co., Ltd., Zhengzhou, China) was applied as base fertilizer at five different rates (0, 40, 70, 100, and 130 kg P_2_O_5_ ha^−1^, denoted as Ck, P1, P2, P3, P4, and P5). Irrigation was conducted twice using a supplementary sprinkler during the establishment year and five times in 2021 and 2022. No diseases or pests occurred during the experiment. Weeds were controlled by mowing or hoeing during the establishment year, as well as in the spring of 2021 and 2022. There were no harvests in the establishment year.

### Sampling, measurements and analysis

#### Sampling and measurements

Overwintering rate: the total number of plants in the quadrat was calculated before and after winter within 1.0 m^2^ randomly selected in each plot. Overwintering rate = (plants after overwintering/plants before winter) × 100%.

Root traits were sampled on October 30, 2020. Ten plants were randomly sampled from each plot, and the entire plant was excavated to facilitate accurate measurement of its root system. The expansion of the crown of the root system was measured with a Vernier caliper and recorded as the crown diameter. The distance from the soil surface to the upper end of the crown is measured with a ruler and recorded as the crown depth. The diameter of the taproot, 1 cm below the crown was measured with a Vernier caliper and recorded as the taproot diameter. The length from the upper end of the taproot to the position where its diameter is 1 mm was also measured using a ruler, and recorded as the taproot length.

Three forage harvests were conducted in both 2021 and 2022. However, due to insufficient regrowth in the spring of 2021, plots T4 and T5 were excluded from sampling. The initial two cuts were carried out at the early flowering stage, while the final cut of each year was scheduled 40 days before the first frost. To ensure accuracy and minimize the edge effect on data, sampling at the borders was avoided. The number of shoots per square meter was counted within a 1.0 m^−2^ in each plot before harvest. A 2.5 m^2^ area from the center of each plot was harvested approximately 5 cm above the ground level to measure the fresh weight. Subsequently, 300 g subsamples of the fresh forage were dried at 105 °C for 30 min followed by 65 °C for 48 h, re-dried, and re-weighed, until a constant weight was achieved, and the dry matter yield per hectare was calculated using the forage fresh weight and the dry-fresh ratio. A subsample of 50 shoots was manually collected from each plot, weighed, and used to calculate the dry mass per shoot by considering the dry-fresh ratio. Taproots within a 1.0 m^2^ area of each plot were excavated to a depth of 10 cm and counted during each harvest to determine the plants per square meter. The annual forage yield was determined by aggregating the yield from all harvests within the year. After each harvest, the border areas surrounding the plots were cleared to maintain the integrity of the experimental setup.

#### Statistical analysis

An analysis of variance (ANOVA) was performed on overwintering rates, root traits, yield, and yield components to evaluate the effects of different treatments. The sowing dates, P treatments, and their interactions were treated as fixed effects, while replication was considered a random effect. The pairwise mean differences were compared using the least significant difference (LSD) with mean differences considered significant at *P* ≤ 0.05^13^. Regression models were developed and evaluated on treatment means, incorporating terms for all significant polynomial contrasts. At times, a regression model displaying an unusual relationship was rejected. The disparities in the slopes of the equations were assessed by employing the standard error of the difference between the slopes, which was adjusted for the experimental error^18^. Statistical analyses were performed using SPSS 26 software, while figures were generated using Origin 2021 software.

### Ethical approval

We confirm that the use of plants in the present study complies with international, national and/or institutional guidelines.

## Results

### Overwintering rates and root traits

Both the sowing dates (T) and P fertilizer significantly influenced overwintering rates and root traits, although the interaction between sowing date and P fertilizer (T × P) was not readily apparent (Table 2). The sowing dates had an extremely significant (*P* < 0.001) impact on the overwintering rates, and highly significant (*P* < 0.01) effects on taproot diameter, crown diameter, and crown depth, and also had a significant (*P* < 0.05) effect on the taproot length. The P fertilization had an extremely significant (*P* < 0.001) effect on the overwintering rates and a highly significant (*P* < 0.01) effect on crown diameter, and a significant (*P* < 0.05) effect on the taproot diameter.

**Table 2:** Level of significance for sowing dates (T), P treatment and T × P interaction effects, and significant orthogonal polynomial contrasts on overwintering rates and root traits.

Source	Overwintering rates	Taproot diameter	Taproot length	Crown diameter	Crown depth
T	***	**	*	**	**
P	***	*	ns	**	ns
T × P	ns	ns	ns	ns	ns

*Significant at the *P* < 0.05 level, **Significant at the *P* < 0.01 level, ***Significant at the *P* < 0.001 level, ns means not significant at the 0.05 probability level. The same as below.

**Figure 1:**
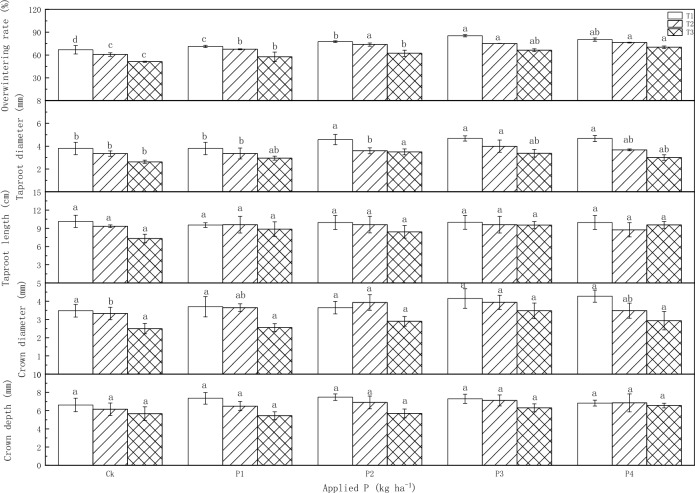
Influence of sowing dates and P application rates on overwintering rates and root traits. Because T4 and T5 had too few regrowth plants in the spring of 2021, which have no further production value, they are not reflected here. The different lowercase letters in the same column mean significant differences among different P application rates on the same sowing date. The same as below.

The study revealed that both the overwintering rates and root traits of alfalfa progressively declined as sowing dates were delayed. However, the response of these variables to P fertilization exhibited varied trends (Fig. 1). In T1, with the increase of P fertilizer, the overwintering rates first increased and then decreased, and the taproot diameter increased, reaching 85.3% and 4.68 mm at P3, respectively. In T2, as P fertilizer levels increased, the overwintering rates went up, and both taproot and crown diameter initially increased and then decreased, where the taproot diameter reached 4.58 mm at P2, and the crown diameter at 3.94 mm at P3 treatment, both significantly different from the control (Ck) at *P* < 0.05. In T3, rising the P fertilizer, the overwintering rates improved, and the taproot diameter initially rose and then fell, reaching 3.50 mm at P2, significantly higher than that of Ck (*P* < 0.05).

**Figure 2:**
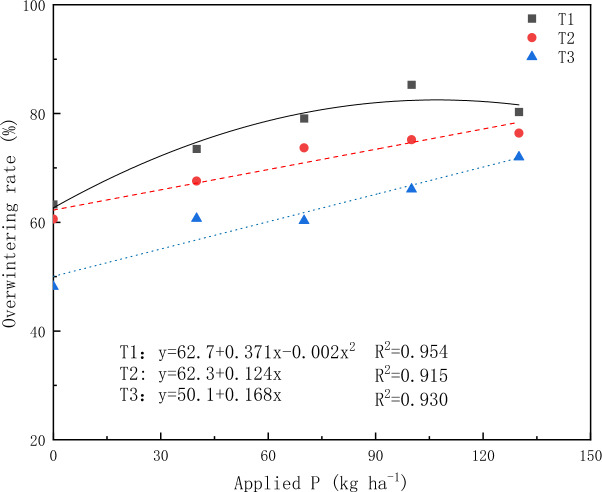
Regression analysis of P application rates on overwintering rates. Each pot means the average of four data.

Curve fitting was generated using the P application rates as the independent variable and the overwintering rates as the dependent variable. A significant (*P* < 0.05) linear regression relationship was found between the overwintering rate and P application rate (Fig. 2). The regression equations for the first, second, and third sowing dates were as follows: y = 62.7 + 0.371x − 0.002x^2^ (R^2^ = 0.954), y = 62.3 + 0.124x (R^2^ = 0.915), and y = 50.1 + 0.168x (R^2^ = 0.930). The overwintering rate of alfalfa in T1 was the highest when the P application rate was 92.8 kg ha^−1^, while the overwintering rate in T2 and T3 increased with the rising P fertilizer application rate.

There were significant correlations between the overwintering rate and various root system indicators (Table 3). The overwintering rate was highly significantly positively correlated with the taproot diameter, taproot length, crown diameter, and crown depth (*P* < 0.01). The taproot diameter exhibited a significant positive correlation with the crown diameter (*P* < 0.01), and highly significant positive correlations with crown depth and taproot length (*P* < 0.05). The taproot length had highly significant positive correlations with the crown diameter and depth (*P* < 0.01). Moreover, the crown diameter and crown depth had a highly significant positive correlation (*P* < 0.01).

**Table 3:** Correlations between overwintering rates and root traits.

Source	Overwintering rates	Taproot diameter	Taproot length	Crown diameter	Crown depth
Overwintering rates	1.000	0.764**	0.716**	0.822**	0.856**
Taproot diameter		1.000	0.626*	0.897**	0.648**
Taproot length			1.000	0.754**	0.679**
Crown diameter				1.000	0.800**
Crown depth					1.000

### Forage yield

Both the sowing dates and P fertilizer affected forage yield, although the interaction (T × P) was not evident (Table 4). In 2021, the sowing dates significantly influenced both the individual harvest and the annual yield, while P fertilizer had a significant impact only on the yield of H3. In 2022, the sowing dates significantly affected the yield of H1, H3, and the annual yield. Additionally, the application of P fertilizer had significant effects on the yield of H1, H2, and annual yield.

**Table 4:** Level of significance for sowing dates (T), P treatment and T × P interaction effects, and significant orthogonal polynomial contrasts on yield, plants m^−2^, shoots m^−2^ and mass shoot^−1^ in 2021 and 2022.

	Source	2021				2022			
		H1	H2	H3	Total	H1	H2	H3	Total
Yield	T	**	**	**	**	**	ns	*	**
	P	ns	ns	*	ns	**	*	ns	**
	T × P	ns	ns	ns	ns	ns	ns	ns	ns
Plants m^−2^	T	**	ns	ns		*	ns	ns	
	P	ns	ns	ns		ns	ns	ns	
	T × P	ns	ns	ns		ns	ns	ns	
Shoots m^−2^	T	ns	ns	ns		ns	**	**	
	P	*	ns	*		ns	ns	**	
	T × P	ns	ns	ns		ns	ns	ns	
Mass shoot^−1^	T	ns	**	ns		**	**	*	
	P	**	**	ns		**	**	**	
	T × P	ns	ns	ns		ns	ns	ns	

H1, H2, and H3 refer to Harvest 1, Harvest 2, and Harvest 3, respectively, the same as below.

**Figure 3:**
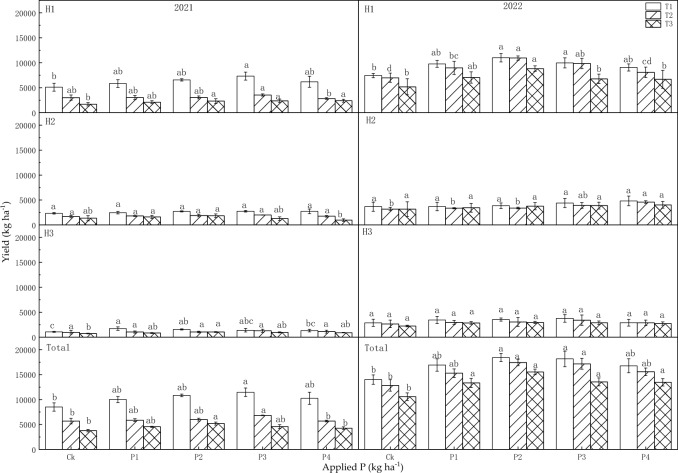
Influence of sowing dates and P application rates on individual harvest and annual forage yield (kg ha^−1^) in 2021 and 2022.

The forage yield of alfalfa exhibited a gradual decrease with delayed sowing dates, while initially increasing and then decreasing with higher levels of P application in both years (Fig. 3). In T1 of 2021, the annual yield at P3 increased by 34.7% compared to the control. In T2 of 2021, the annual yield at P3 significantly increased by 20.2% compared to the control. In T3 of 2021, the annual yield increased significantly at P2 by 37.0% compared with the control. In T1 of 2022, there was a significant increase in the annual yield at P2 and P3, increasing by 31.8% and 29.7% compared to the controls. In T2 of 2022, the annual yield significantly increased at P2 and P3 by 35.8% and 33.8% compared to the control. In T3 of 2022, the annual yield significantly increased when P applied, by more than 26.0% compared to the control. The annual yield of the last two sowing periods decreased by 41.1% and 56.1%, respectively, compared to that of the first sowing period in 2021, and decreased by 7.1% and 21.1% in 2022, without considering the impact of P fertilizer.

**Figure 4:**
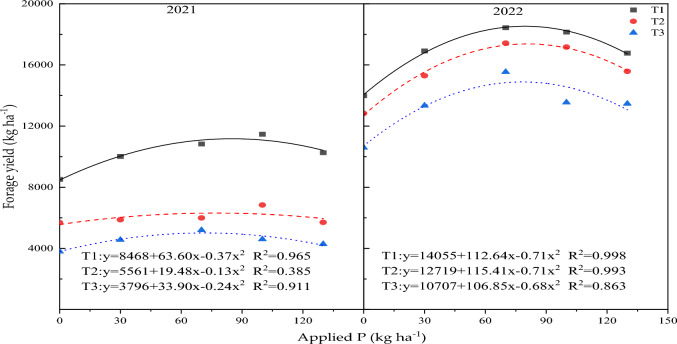
Regression analysis of P application rates on forage yield in each harvest of 2021 and 2022. Each pot means the average of four data.

The results of curve fitting indicated linear quadratic regression associations between forage yield and P application rate (Fig. 4). In 2021, the regression equations for the first, second, and third sowing dates were as follows: y = 8468 + 63.60x − 0.37x^2^ (R^2^ = 0.965, *P* < 0.05), y = 5561 + 19.48x − 0.13x^2^ (R^2^ = 0.385, *P* > 0.05), and y = 3796 + 33.90x − 0.24x^2^ (R^2^ = 0.911,* P* > 0.05), respectively. The recommended range for the P application rate was between 70.6 kg ha^−1^ and 85.9 kg ha^−1^. In 2022, the regression equations were as follows: y = 14,055 + 112.64x − 0.71x^2^ (R^2^ = 0.998, *P* < 0.01), y = 12,720 + 115.41x − 0.72x^2^ (R^2^ = 0.993, *P* < 0.01), and y = 10,707 + 106.85x − 0.68x^2^ (R^2^ = 0.863, *P* > 0.05). The suitable range for the amount of P application rate was between 78.6 and 80.1 kg ha^−1^.

### Yield components

#### Plant population density

The sowing dates had a very significant impact on the number of plants per square meter of H1 in 2021 (*P* < 0.01) and 2022 (*P* < 0.05), but P fertilizer and the interaction (T × P) did not exhibit a clear effect on the plant population density (Table 4).

**Figure 5:**
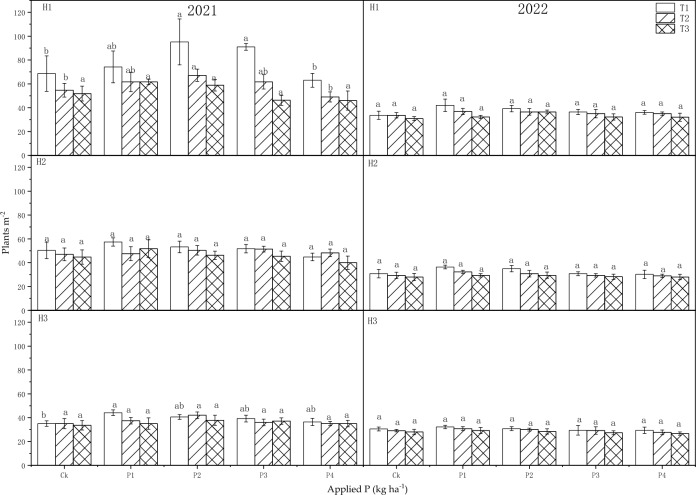
Influence of sowing dates and P application rates on plants m^−2^ in 2021 and 2022.

The number of plants per square meter of alfalfa exhibited a gradual decline with delayed sowing dates, while it initially increased and then decreased with higher levels of P application in both years (Fig. 5). In 2021, the plants per square meter at P2 and P3 were significantly greater than that of the control (Ck) in H1 of T1 (*P* < 0.05). Additionally, compared to the Ck, the plants per square meter was significantly increased at P1 level in H3 (*P* < 0.05). In T2, the plants per square meter at P2 level was significantly greater than Ck in H1 (*P* < 0.05). In 2022, the application of P fertilizer had no significant impact on plants per square meter. The plants per square meter of three sowing dates decreased by 61.1%, 49.8%, and 47.4%, respectively from H1 of 2021 to H3 of 2022.

#### Shoots per square meter

The sowing dates and P fertilizer both significantly impact the shoots per square, although their interaction did not show an obvious effect (Table 4). In 2021, the P application had a significant impact on shoots per square meter at H1 and H3. In 2022, the sowing dates had a very significant effect on shoots per square meter at H2 and H3 (*P* < 0.01). Moreover, P fertilizer application also significantly affected the shoots per square meter at H3 (*P* < 0.01).

**Figure 6:**
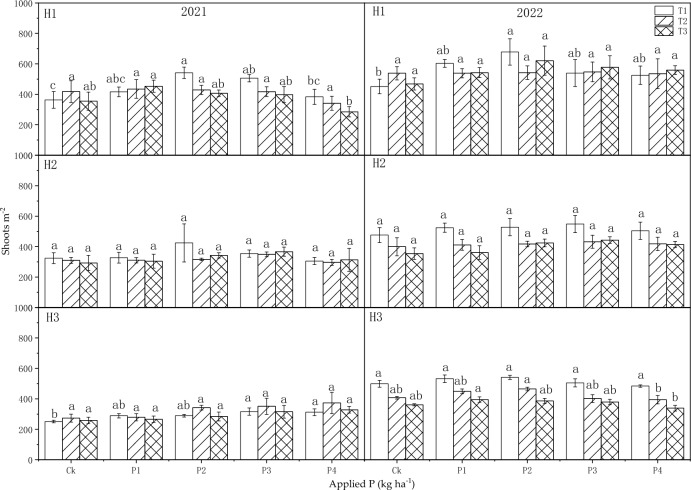
Influence of sowing dates and P application rates on shoots m^−2^ in 2021 and 2022.

The relationship between shoots per square meter and the sowing dates did not show a clear trend. However, with increasing the P fertilizer application rate, the shoots per square meter first increased and then decreased (Fig. 6). In 2021, the shoots per square meter was 441 in T1, significantly greater than that in T3 (*P* < 0.05). Compared to the Ck, the shoots per square meter were significantly greater in H1 at P2 and P3, and in H3 at P3 and P4 in T1 (*P* < 0.05). In 2022, the shoots per square meter was significantly higher than that of the control in H1 at P2 in T1 (*P* < 0.05).

#### Mass per shoot

The sowing dates and P fertilizer both influenced the mass per shoot, although their interaction (T × P) did not show an obvious effect (Table 4). In 2021, the sowing dates had a very significant influence on the mass per shoot in H2 (*P* < 0.01); P fertilizer also had a very significant impact on the mass per shoot in H1 and H2 (*P* < 0.01). In 2022, the sowing dates influenced mass per shoot in H1, H2 (*P* < 0.01), and H3 (*P* < 0.05) significantly. Furthermore, P fertilizer had a very significant effect on mass per shoot at each harvest (*P* < 0.01).

**Figure 7:**
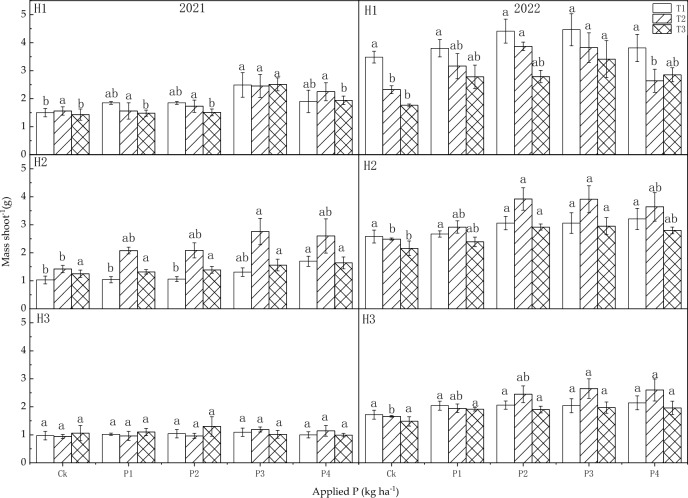
Influence of sowing dates and P application rates on mass shoot^−1^ in 2021 and 2022.

The trends of mass per shoot varied with the delayed sowing dates and the application of P fertilizer (Fig. 7). In 2021, the mass per shoot initially increased and then decreased with the application of P fertilizer in H1 of T1. At P3, the mass per shoot was 2.49 g which was higher than that of the control (*P* < 0.05). The mass per shoot increased with the application of P fertilizer, reaching 1.70 g at P4, significantly higher compared to the control (*P* < 0.05) in H2 of T1. The mass per shoot first increased and then decreased with the increase of P application in H2 of T2 and H1 of T3, reaching 2.76 g and 2.51 g at P3, respectively, which were greater than the controls (*P* < 0.05). In 2022, the application of P did not influence mass per shoot at any harvest significantly in T1. The mass per shoot first increased and then decreased with the application of P in H1 of T2, reaching 3.87 g and 3.82 g at P2 and P3, respectively, which were significantly greater than that of the control (*P* < 0.05). Similarly, the mass per shoot increased with the application of the P fertilizer rate in H3 of T2, reaching 2.65 g at P4. The mass per shoot first increased and then decreased with the application of P fertilizer in H1 and H2 of T3. Specifically, the masses per shoot were 3.41 g at P2 in H1, and 2.95 g at P3 in H2, respectively, which were greater than that of the controls (*P* < 0.05).

### Regression analysis of yield components on forage yield

Linear relationships between forage yield and shoots per square meter and mass per shoot were examined using regression analysis (Table 5). Notably, significant linear relationships between forage yield and shoots per square meter were observed 4 out of 18 times over 2 years. The R^2^-values ranged from 0.00 (Harvest 3 of T1 in 2021) to 0.32 (Harvest 2 of T3 in 2022). Although the R^2^-values were relatively low, the positive slopes indicate that increases in shoots per square meter led to higher forage yield at these specific harvests for all sowing dates, except for H3 in T1 of 2021. The relationship between mass per shoot and forage yield demonstrated positive linear relationships 7 out of 18 times over 2 years. The R^2^-values ranged from 0.02 (H1 of T2 and H3 of T3 in 2021) to 0.53 (H3 of T3 in 2022), with R-value exceeding 0.40 in 5 out of 18 times, suggesting a correlation between the variation in forage yield and mass per shoot. Conversely, the R^2^-values derived from the regression analysis of shoots per square meter on forage yield were consistently below 0.40 across all harvests.

**Table 5:** Regression equations, R^2^-values, and P-values for the influence of shoots m^−2^ and mass shoot^−1^ (g) on forage yield at each harvest in 2021 and 2022.

Sowing dates	Harvests	Shoots (no. m^−2^)	R^2^	*P*-value	Mass shoot^−1^ (g)	R^2^	*P*-value
	2021						
T1	H1	Y = 2626 + 8.10x	0.27	0.019	Y = 2703 + 1827.58x	0.46	0.001
	H2	Y = 2507 + 0.28x	0.01	0.636	Y = 2310 + 238.72x	0.09	0.210
	H3	Y = 1445–0.11x	0.00	0.956	Y = 960 + 442.23x	0.10	0.165
T2	H1	Y = 2600 + 1.22x	0.09	0.201	Y = 2950 + 76.28x	0.02	0.601
	H2	Y = 934 + 2.82x	0.08	0.215	Y = 1345 + 219.14x	0.29	0.015
	H3	Y = 705 + 1.22x	0.18	0.065	Y = 765 + 321.69x	0.10	0.166
T3	H1	Y = 2025 + 0.38x	0.01	0.702	Y = 1735 + 246.09x	0.10	0.181
	H2	Y = 904 + 1.58x	0.10	0.183	Y = 1763 + 243.88x	0.03	0.471
	H3	Y = 818 + 0.28x	0.01	0.704	Y = 835 + 58.47x	0.02	0.569
	2022						
T1	H1	Y = 7537 + 3.43x	0.07	0.256	Y = 6898 + 640.64x	0.09	0.202
	H2	Y = 2231 + 3.61x	0.15	0.096	Y = 2832 + 432.13x	0.08	0.237
	H3	Y = − 455 + 7.35x	0.20	0.047	Y = 2384 + 462.91x	0.07	0.270
T2	H1	Y = 6842 + 3.95x	0.07	0.273	Y = 5070 + 1234.95x	0.47	0.001
	H2	Y = 1512 + 5.25x	0.25	0.023	Y = 2076 + 479.04x	0.29	0.014
	H3	Y = 1134 + 4.40x	0.08	0.243	Y = 1265 + 765.92x	0.45	0.001
T3	H1	Y = 4107 + 5.05x	0.13	0.116	Y = 5390 + 555.95x	0.09	0.212
	H2	Y = 715 + 7.37x	0.32	0.010	Y = 564 + 1170.90x	0.42	0.002
	H3	Y = 737 + 5.38x	0.13	0.126	Y = 715 + 1096.90x	0.53	< 0.000

Two-way path analysis revealed that mass per shoot generally had a greater impact on forage yield compared to shoots per square meter (Table 6). The path coefficient ratios of mass per shoot to shoots per square meter were greater than 1.0 in 12 of 18 harvests. This ratio exceeded 1.0 in 4 out of 6 harvests for each sowing date, suggesting that the sowing dates did significantly influence the relative importance of the main yield components.

**Table 6:** Path coefficient analysis at each harvest in 2 years.

Sowing dates	Two-way path analysis	2021			2022		
		H1	H2	H3	H1	H2	H3
T1	Shoots m^−2^ → yield	0.325	0.195	0.002	0.315	0.390	0.432
	Mass shoot^−1^ → yield	0.568	0.340	0.323	0.343	0.286	0.225
	Joint effect of mass shoot^−1^ and shoots m^−2†^	0.304	0.129	0.011	0.093	0.016	0.053
	Mass shoot^−1^: shoots m^−2^	1.75:1	1.74:1	161.5:1	1.09:1	0.73:1	0.52:1
T2	Shoots m^−2^ → yield	0.397	0.231	0.355	0.037	0.423	0.207
	Mass shoot^−1^ → yield	0.269	0.509	0.217	0.698	0.465	0.652
	Joint effect of mass shoot^−1^ and shoots m^−2†^	0.243	0.087	0.170	0.309	0.155	0.088
	Mass shoot^−1^: shoots m^−2^	0.68:1	2.20:1	0.61:1	18.86:1	1.10:1	3.15:1
T3	Shoots m^−2^ → yield	0.108	0.374	0.146	0.357	0.331	0.176
	Mass shoot^−1^ → yield	0.318	0.262	0.180	0.284	0.491	0.678
	Joint effect of mass shoot^−1^ and shoots m^−2†^	0.023	0.155	0.100	0.013	0.388	0.224
	Mass shoot^−1^: shoots m^−2^	2.94:1	0.70:1	1.23:1	0.80:1	1.48:1	3.85:1

^†^Joint effect is also referred to as the compound or indirect path (example: mass shoot^−1^ ↔ shoots m^−2^ → yield) and calculated as the product of two individual paths.

## Discussion

### Effect on the overwintering rates and root traits

With autumn and winter approaching, the temperature drops gradually, and perennial crops like alfalfa enter the dormancy period, stop growing to resist adverse environmental conditions, and survive the winter safely^22,23^. The earlier the alfalfa is sown before winter, the alfalfa can obtain more suitable climatic conditions, thereby shortening the germination time and prolonging the period for vegetative growth, allowing for a more comprehensive root system development, storing sufficient nutrients and energy storage for overwintering and growing in the following year^7,24^. Alfalfa plants must have 2 to 3 branches in the establishment year before winter to ensure their safe overwintering^20^. Sun et al.^25^ believed that the improper sowing date leads to insufficient root growth and shallow crown of alfalfa, so the suitable sowing date will improve the overwintering rate of alfalfa^31^. Our study showed that delaying the sowing date progressively impairs the root traits: taproot and crown diameters became smaller, and crown depth became shallower to the soil, leading to reduce the overwintering rate of alfalfa. When alfalfa was sown later than mid-August, the number of green plants in spring was too low and it had no production value. This decline was attributed to the reduced growth and development period available to alfalfa seedlings before winter, limiting root growth and root carbohydrate accumulation^26,27^. However, the application of P fertilizer can promote the alfalfa root system development and improve the energy storage within the root system before winter, thereby enhancing the alfalfa cold resistance and improving production performance^13,27^. Our study observed that an appropriate application of P fertilizer encouraged the growth of late-seeded alfalfa root traits, especially the taproot and crown diameter, thereby enhancing the overwintering rates. Although late sowing affected the root development of alfalfa, the application of P fertilizer can promote the storage of energy and nutrient recovery in the root, promoting root system development, and enhancing the plant's ability to survive the winter^28^.

### Effect on the forage yield

The selection of suitable sowing time for alfalfa is a crucial technical aspect of its cultivation, which changes the water and temperature faced by alfalfa seedlings during their growth stage^7^, as it has a significant impact on seedling management, growth and development, persistence, and stress resistance^29^. Teixeira et al.^5^ reported a similar yield reduction with delayed sowing from early spring to late summer. Our study aligns with these findings, the alfalfa forage yield decreases progressively with delayed sowing, the earlier the sowing, the more beneficial it is for the growth of alfalfa the following year. The lower limit of the sowing date threshold for autumn sowing in the experiment site is mid-August, if alfalfa sown after mid-August failed to regenerate adequately in the second year, halting it unproductive^30^.

Phosphorus application is an important agronomic practice for enhancing forage yield in alfalfa, due to the high P requirements of the crop^31^. Wang et al.^32^ reported that alfalfa yield increased with higher P application rates, but the optimal P application rate varied across different regions. James et al.^33^ reported that in the intermountain western United States, an application of 220 kg P ha^−1^ was sufficient to maintain a high yield for at least 3 years. Xie et al.^34^ found that in Hebei Province, China, the application of 225 kg ha^−1^ P fertilizer led to a 67.94% increase in alfalfa yield. In our study, with the increase in P application rate, the forage yield increased first and then decreased. The effect of applying P fertilizer in the second production year was particularly significant, and the forage yield of alfalfa reached 15,542 kg ha^−1^ when 70 kg P ha^−1^ was applied during the third sowing period, which was much higher than the forage yield when no fertilizer was applied during the first sowing period. The regression analysis concluded that the optimal P application rates ranged from 70.6 to 85.9 kg ha^−1^ in 2021 and from 78.6 to 80.1 kg ha^−1^ in 2022. Applying P fertilizer to the late-seeded alfalfa, the forage yield of the second year would not be less than that of the earlier sowing. It should be noted that the reduction in yield caused by late sowing can be compensated by fertilization, and alfalfa sowing in August can be performed after the harvest of a short-season crop, the lower production annual yield may be compensated for by the value of the short-season crop^35^.

### Effect on the yield components

Alfalfa forage yield can be described as the product of three components: plants per area, shoots per plant, and mass per shoot^36^. Plants per area and shoots per plant collectively determine the number of shoots per area, so forage yield is ultimately determined by shoots per area and mass per shoot. If the sowing time is too late, the air temperature drops greatly, especially at night, which may seriously affect the growth of plant seedlings. These seedlings tend to be smaller and mostly single-branched, resulting in weaker cold resistance and only a few plants regrowing in the subsequent year^7^. In our study, the plant density gradually decreased with the delay in sowing time and the plant density decreased with the increase of cutting times. Which were with a more pronounced decrease in the first year, which gradually diminished in the second year. Stress factors such as defoliation and diseases, along with competition for water, light, and nutrients may lead to plant death in summer. Moreover, winter-injured plants may survive to be counted in June but may ultimately succumb over the summer, due to a misperception that plant loss reflects summer-related stresses^18^. In our study, the plant density initially increased and then decreased with the application of P fertilizers, which is consistent with our previous research results^13^. The application of P fertilizer can promote the growth of plant roots, and increase the surface area and volume of the root system, thereby enhancing the plant’s ability to absorb water and nutrients, which in turn can increase the plant’s stand density^37^. However, Berg et al.^18^ found that the application of P fertilizer decreased the plant density due to the intensified intraspecific competition. This discrepancy could be related to the alfalfa varieties used and the initial P levels in the soil. Moreover, the overall sowing date of this experiment is relatively late, and the initial plant density was already low, resulting in weak competition for light, water, and nutrients. Therefore, the application of P fertilizer positively influenced plant density.

Alfalfa yield has been positively associated with shoots per square meter, and this yield component is often as a criterion to determine whether or not to keep an existing alfalfa stand^19^. The prediction of alfalfa yield potential often relies on shoots per area, with significant losses in forage yield anticipated when shoot density declines below the “benchmark” value of 430 shoots m^−2^ (40 shoots ft^−2^)^38^. In our study, the shoots per square meter did not vary between sowing dates in 2021, but decreased with the delay of sowing dates in H2 and H3 of 2022. Although the plant density of alfalfa changed in the early stage, alfalfa was also branching with growth and development, so there was no significant change in the number of shoots per square meter^24,39^. However, in the later stage, with the further decrease of the plant density, the increase of shoots per plant is not enough to make up for the decrease of shoots per square meter. Regression analysis of shoots per square meter versus forage yield revealed significant linear relationships between these two traits at 4 of 18 harvests, indicating that higher forage yields are not invariably associated with more shoots per square meter^19^. Thus, using this yield component to estimate the future yield potential of alfalfa may not always be suitable^18^. After applying P fertilizer, the shoot growth of alfalfa can be greatly improved, to increase the forage yield^40^. Suzuki and Michio^39^ reported that the shoots per square meter and forage yield can be maintained at a high level with a reasonable level of P and potassium fertilizer. In our study, the number of shoots per square meter initially increased and then decreased in only 6 of 18 harvests, almost in H1 and H3 of each year. It can be seen that the application of P fertilizer can increase the shoots per square meter to some extent. This may be due to the application of P fertilizer significantly increased the utilization efficiency of starch stored in taproot and root crown into regenerated biomass and promoted the germination of dormant buds and the growth of new shoots, thus increasing the number of shoots per plant^41^.

Mass per shoot is also another important component of forage yield^18,19,42^. Increased mass per shoot has been positively associated with enhanced forage yield of alfalfa, whether differences were caused by improved genetics, soil fertility, or insect control^43^. Tian^44^ found that with delayed sowing dates, the mass per shoot of alfalfa exhibited varying trends among varieties and harvests. In our study, postponement of sowing dates significantly affected the mass per shoot 4 of 6 times, showing an initial increase and subsequent decrease in H2 of 2021, H2 and H3 of 2022; and a decrease in H1 in 2022; respectively. Plants that were fertilized exhibited a higher mass per shoot compared to those that were not, P fertilizer additions increase mass per shoot via two mechanisms: rapid initiation of shoot regrowth after defoliation and increased shoot growth rate between harvests^45^. Lu et al.^46^ reported that P fertilizer treatment significantly increased the weight of shoots from the root crown and stem of alfalfa in each harvest. In our study, the mass per shoot increased with the application of P fertilizer, the greatest mass per shoot was 4.46 g at P3 in H1 of 2022. Regression analysis of mass per shoot versus forage yield revealed significant linear relationships between these two traits in 7 of 18 harvests. Path coefficient analysis showed that the mass per shoot made more contribution to forage yield than shoots per square meter. Therefore, phosphorus fertilizer mainly improved forage yield by increasing mass per shoot, and the reason is that P promoted the nutrient flow between roots and new shoots, thereby accelerating the growth of the new shoots^46^.

### Application prospect

The central and western parts of Inner Mongolia belong to a typical temperate continental climate with cold and long winters, which presents a unique challenge for crop cultivation. The climate dictates that planting one crop per growing season is feasible, but attempting to grow two crops within the same period is not viable due to the insufficient time for the second crop to mature fully^47^. The success of our research paved the way for an innovative farming strategy: harvesting a short-season crop like oats or corn before sowing alfalfa. This approach would not only maximize the use of the growing season but also enhance the economic returns for farmers without compromising the productivity of the perennial alfalfa in subsequent years. The potential increase in income could significantly boost the morale and motivation of farmers to cultivate alfalfa. Moreover, the integration of such a cropping system has a profound impact on soil health by improving soil fertility, and structure, and reducing the build-up of pests and diseases^48^. It also allows for the replenishment of different nutrient levels in the soil, as different plants have varying nutrient requirements and contribute different residues back to the soil. Additionally, this cropping system can help in the management of soil erosion by maintaining or enhancing soil structure through the root systems of different plants^49^.

The economic implications of this research are profound, as it could lead to a paradigm shift in agricultural practices within regions with similar climatic conditions. By optimizing the use of land and extending the harvest window, farmers could see a notable increase in their annual income, which would not only improve their quality of life but also contribute to the overall food security and agricultural sustainability of the region.

## Conclusions

In arid and cold regions, the overwintering capability and productivity of alfalfa progressively decreased with delayed sowing. The decline in overwintering rate is largely attributed to a reduction in root traits, whereas the decrease in forage yield mainly results from fewer plants per square meter. However, applying P fertilizer to late-seeded alfalfa can enlarge the diameter of the crown and taproot, thereby, strengthening the root robustness and improving the overwintering rate, the rate of increase ranges from 11.6 to 49%. This adjustment could also lead to an increase in shoots per square meter and mass per shoot, increasing by 9.4–31.3% and 15.0–27.1% respectively in 2 years, which can offset the decline in forage yield caused by late sowing and might even increase the forage yield. The findings from regression and path analyses indicate that the alfalfa yield is more significantly influenced by mass per shoot rather than shoots per square meter. It is recommended that the alfalfa sowing in similar regions of Inner Mongolia should not be postponed beyond mid-August. Moreover, the application of P fertilizer (P_2_O_5_) in the range of 70.6–85.9 kg ha^−1^ can enhance the forage yield and persistence of late-seeded alfalfa. Therefore, appropriate late sowing combined with the application of P fertilizer can be used as an efficient cultivation strategy for alfalfa cultivation after a short-season crop harvest in arid and cold regions.

## Data Availability

The datasets generated during and/or analyzed during the current study are available from the corresponding author or the first author upon reasonable request.
